# *Bletilla striata* Polysaccharide: Structural Characteristics, Chemical Modification Strategies, and Dual-Field Application Progress in Biomedicine and the Food Industry

**DOI:** 10.3390/molecules31101640

**Published:** 2026-05-13

**Authors:** Fei Wang, Jingyuan Peng, Zangjia Geng, Shanshan Li, Xuemei Yin, Xueting Feng, Zhichao Jiang, Yaoyu Xiao, Kun Guo, Xiaoling Wang

**Affiliations:** 1 College of Pharmacy and Food, Southwest Minzu University, Chengdu 610225, China; 18708898349@163.com (F.W.); pjy20030307@163.com (J.P.); 21800007@swun.edu.cn (Z.G.); sslscu2009@163.com (S.L.); ucasyinxm@163.com (X.Y.);; 2 National Base for International Science and Technology Cooperation, School of Pharmacy, Chengdu University, Chengdu 610106, China; 3 State Key Laboratory of Polymer Materials Engineering, School of Mechanical Engineering, Sichuan University, Chengdu 610065, China; zhichao.jiang@scu.edu.cn (Z.J.); yaoyu.xiao@scu.edu.cn (Y.X.)

**Keywords:** *Bletilla striata* Polysaccharide, structural characterization, chemical modification, biomedical application, food applications, structure–activity relationship

## Abstract

*Bletilla striata* polysaccharide (BSP), a bioactive glucomannan derived from the traditional Chinese medicinal herb *Bletilla striata*, has garnered increasing attention in both the biomedical and food sectors due to its unique physicochemical properties and diverse biological activities. While existing reviews have partially covered BSP’s structural features or single-field applications, a systematic review integrating its structure–activity relationship, full-spectrum chemical modification strategies, and parallel advances in the dual core fields of biomedicine and the food industry remains lacking. This review systematically consolidates recent advances in BSP research, focusing on three interconnected aspects: (1) the structure–activity relationships of BSP, highlighting how molecular weight (10^4^–10^5^ Da), monosaccharide composition (mainly glucose and mannose with variable ratios), glycosidic linkages, and higher-order self-assembled structures (e.g., triple-helix conformation) dictate its functionality in biological systems and food matrices; (2) chemical modification strategies—including carboxymethylation, graft copolymerization, cross-linking, polysaccharide–trace element complexation, phosphorylation, acetylation, and cholesterylation—that overcome intrinsic limitations of native BSP to enhance solubility, targeting, bioactivity, and food-related functional properties; and (3) the expanding applications of BSP and its derivatives in biomedicine (hemostatic materials, tissue engineering scaffolds, drug delivery systems, immunomodulation, and antitumor effects) and in the food industry (as natural stabilizers, emulsifiers, functional additives, and bio-based packaging components). Compared with previously published reviews, this work establishes a complete closed-loop logical system from structural characterization to rational modification and cross-field application and provides the most up-to-date systematic summary of BSP research. Key challenges—such as an incomplete understanding of structure-function correlations, insufficient pharmacokinetic data, and a lack of standardized quality control—are discussed, and future research directions are proposed. This review aims to provide a systematic theoretical basis for advancing BSP as a versatile multifunctional material for applications in functional foods, nutraceuticals, and biomedical fields.

## 1. Introduction

*Bletilla striata* is a traditional Chinese medicinal herb, also known as “Baiji” or “Dilan” in China. It belongs to the genus *Bletilla* of the Orchidaceae family and is an endangered perennial herbaceous plant [1,2]. From the perspective of TCM (traditional Chinese medicine) meridian tropism theory, *B. striata* mainly acts on the lung, liver, and stomach meridians. It can be processed into a dietary supplement with the effects of nourishing lung yin and invigorating the liver and kidneys [3]. *B. striata* contains a variety of important bioactive substances, mainly including dibenzyls, phenanthrenes, polysaccharides, flavonoids, anthocyanins, steroids, triterpenes, esters, saponins, and trace elements [4,5,6]. Among these active components, BSP serves as the key material basis for *B. striata* to exert its biological activities. It exhibits multiple pharmacological effects, such as immunomodulation [7,8], antioxidant activity [9], gastrointestinal protection [10,11], hepatoprotection [12], anti-inflammation [13,14], antibacterial activity [15], antitumor effect [5] and hemostatic activity [16].

BSP possesses unique physicochemical properties and biological activities, exhibiting extremely broad application potential and development prospects in the food industry and medicine. For instance, its excellent biocompatibility, biodegradability, and adhesiveness make it a high-quality carrier option for precise drug delivery and controlled release. Ranging from wound repair to chronic disease treatment and from improving the body’s microecology to regulating overall physiological functions, BSP is gradually becoming a research focus in numerous scientific fields. However, existing studies lack systematic exploration of *Bletilla striata* varieties, extraction methods, structural characterization, and structure–activity relationship analysis, with insufficient coverage in application fields.

Compared with industrially established natural polysaccharides, BSP exhibits distinct advantages. Chitosan is limited by poor solubility at neutral pH [17]. Hyaluronic acid lacks intrinsic bioactivity and degrades rapidly [17]. Sodium alginate hydrogels are mechanically fragile and bioinert [18]. Even konjac glucomannan—structurally analogous to BSP—has a higher molecular weight, excessive viscosity, and no tissue-adhesive or hemostatic function [3]. In contrast, BSP uniquely integrates: (1) excellent water solubility across a wide pH range without derivatization; (2) a flexible, modifiable backbone enabling film-forming, adhesion, and chemical tailoring; (3) innate hemostatic, anti-inflammatory, and antioxidant bioactivities; and (4) a “medicine–food homology” status ensuring regulatory compliance in food applications. BSP thus functions as an integrated bioactive material rather than a mere structural excipient.

This review is premised on the hypothesis that the biofunctionality of BSP is fundamentally governed by its definable structural parameters, molecular weight, branching, and higher-order conformation. We further postulate that rational chemical modification serves as a critical strategy to tailor these inherent properties, thereby amplifying and steering their performance for targeted applications in biomedicine and functional foods, a correlation systematically examined here through the integration of recent advances (2016–2025). Notably, this work distinguishes itself from existing reviews in three core aspects: (1) it constructs a complete logical chain of “structure–activity relationship–chemical modification regulation–dual-field application”, addressing the fragmented narrative of previous reviews; (2) it provides the first systematic and parallel summary of BSP’s full-dimensional advances in both biomedicine and the food industry, filling the gap of single-field focus in existing works; (3) it primarily covers the research progress of BSP over the past decade (2016–2025), with a particular focus on the most recent advances from 2021 to 2025. While foundational studies published before 2021 are included to provide essential structural and mechanistic context, the core analysis and application examples are drawn predominantly from the 2021–2025 literature.

## 2. Structure–Activity Relationship of *Bletilla striata* Polysaccharide

From a chemical perspective, the biological functions of BSP are closely associated with its structural characteristics, and this structure–activity relationship is primarily reflected in multiple dimensions, including molecular weight, monosaccharide composition, glycosidic bond type, and higher-order structure [19]. First, molecular weight not only affects the physicochemical properties and biological activities of BSP but also directly influences its pharmaceutical applications. However, it should be noted that different detection methods may lead to variations in measured molecular weight values. Second, structural heterogeneity manifests as differences in the monosaccharide ratio, glycosidic bond linkage, and branched structure, all of which collectively determine BSP’s biological activity. More importantly, BSP can form higher-order structures through intramolecular or intermolecular interactions; this self-assembly behavior endows BSP with unique functional properties, laying a structural foundation for its biomedical applications. Therefore, a clear structure–activity relationship exists between BSP’s structural characteristics and biological activities, and changes in structural parameters can result in significant differences in its functional performance.

### 2.1. Composition and Structure of Bletilla striata Polysaccharide

Multiple studies have confirmed that BSP is a glucomannan [20,21]. As early as 1993, a study first confirmed via chemical analysis that the BSP backbone consists of glucose (Glc) and mannose (Man) residues linked by β-(1→4) glycosidic bonds [22]. Compared with other glucomannans, *Bletilla striata*-derived glucomannan is distinctive in its unique monosaccharide ratio, specific glycosidic bond type, and clear primary structure. Affected by the *Bletilla striata* variety and extraction processes, the monosaccharide composition of BSP exhibits significant heterogeneity. For example, BSAP (22.9 KDa) is composed of Man, Glc, and xylose (Xyl), differing from classic BSP (which contains galactose, Gal) [23].

Existing research has uncovered diverse BSP structural subtypes. BSP-1 and BSP-2, for instance, are primarily composed of Man and Glc, with molar ratios of 4.0:1.0 and 3.0:1.0, and average molecular weights (MWs) of 83.54 KDa and 12.6 KDa, respectively. Spectroscopic analysis reveals both have linear backbones of β-(1→4)-linked D-Man*p* and D-Glc*p*, though BSP-1 exhibits a broader MW distribution [24]. BSPF2 exhibits more complex structural characteristics: its monosaccharide ratio is Man:Glc:Gal = 9.4:2.6:1.0; its backbone consists of (1→4)-linked Man*p* and Glc*p* residues, with approximately 60% of Glc*p* residues branched at the O-6 position; and its terminal sugar residues are Man*p* [25]. Differences in glycosidic bond configurations also exist among BSP from different sources. The backbone of BSPb consists of (1→4)-linked α-(1→4)-linked D-Man*p* and β-(1→4)-linked D-Glc*p* [26]. The Man: The Glc molar ratio of RBP is 2:1; its backbone structure is similar to that of BSP-1 and BSP-2 but with a lower degree of branching [27]. A water-soluble polysaccharide (pBSP) extracted from *Bletilla striata* was analyzed via Fourier Transform Infrared Spectroscopy (FT-IR), Scanning Electron Microscopy (SEM), and Nuclear Magnetic Resonance (NMR). The results showed that its backbone consists of β-(1→4)-linked D-Man*p*, α-(1→4)-linked D-Glc*p*, and β-(1→3)-linked D-Man*p*, with no branched structures detected [28]. Further studies have revealed the fine structural characteristics of BSP. Chen et al. [29] analyzed the chemical structure of a novel polysaccharide (pFSP), a homogeneous polysaccharide composed of Glc, Gal, and Man with a molar ratio of 1.00:2.03:3.45. Its repeating unit contains α-(1→4)-linked D-Glc*p*, β-(1→4)-linked D-Man*p*, and β-(1→3,6)-linked D-Man*p*, with β-(1→6)-linked D-Gal*p* branches terminated by β-(1→)-linked D-Man*p* residues. Chen et al. [9] prepared BSPs (e.g., BSPs-H, BSPs-A) via different extraction processes: BSPs-A showed excellent DPPH• and ABTS radical scavenging abilities, while BSPs-H exhibited better hydroxyl radical scavenging efficiency. This difference is closely related to BSP’s rough lamellar structure and α-glucopyranose conformation. The degree of branching of BSP significantly affects its antioxidant activity. For example, BSPF2 (Man:Glc:Gal = 9.4:2.6:1.0) with ~60% branching at the O-6 position of Glc*p* residues exhibits 2.3-fold higher DPPH• scavenging activity than linear pBSP [9]. This is attributed to the loose chain structure of highly branched BSP, which exposes more hydroxyl groups as free radical scavenging sites. In addition, pFSP (with a higher degree of branching) exhibits stronger antioxidant activity than pBSP, attributed to its loose chain structure and increased exposure of active sites [29]. Structural differences among different BSPs arise from variations in *Bletilla striata* sources, extraction methods, and glycosidic bond configurations; the analysis of their fine structures provides a basis for studying functional activities.

### 2.2. Molecular Weight

Molecular weight (MW) is a critical parameter of BSP. Variations in MW significantly alter BSP’s physicochemical properties, biological activities, and in vivo processes—ultimately impacting its performance as a drug carrier and the stability of pharmaceutical formulations [30]. Current techniques for determining BSP’s MW include gel permeation chromatography (GPC) [9], high-performance liquid chromatography (HPLC) [23], and high-performance gel permeation chromatography (HPGPC, a term synonymous with high-performance size exclusion chromatography, HPSEC) [31]. Among these, HPGPC (HPSEC) is the most widely used for analyzing BSP’s molecular weight distribution [24]. When coupled with multi-angle laser light scattering and refractive index detection (MALLS-RID), HPGPC (HPSEC) can accurately measure BSP’s weight-average molecular weight, number-average molecular weight, and polydispersity index. Huang et al. [20] isolated and purified a novel neutral polysaccharide from *B. striata* with a weight-average MW of 28,365 Da, while Lin et al. [21] obtained a new BSP variant via a low-temperature vacuum system, with an MW of 2.35 × 10^5^ Da. These findings provide key data for characterizing BSP’s MW distribution, and existing studies consistently place BSP’s MW in the range of 10^4^–10^5^ Da [3]. Notably, raw material sources, extraction methods, and detection techniques significantly affect BSP’s molecular weight. For example, compared with hot water extraction (HWE), ultrasound-assisted extraction (UAE) yields BSP with a smaller molecular weight, and the molecular weight further decreases with increasing ultrasonic power [9]. Therefore, in practical research, appropriate detection methods should be reasonably selected based on specific research needs and experimental conditions to ensure the reliability of molecular weight measurement results. Multiple studies have confirmed that the molecular weight and glycosidic bonds of BSP are significantly correlated with its biological activities [32,33]. For instance, compared with low-molecular-weight BSP-2 (12.60 KDa), high-molecular-weight BSP-1 (83.54 KDa) exhibits a stronger ability to induce macrophages to release nitric oxide, reactive oxygen species, and inflammatory cytokines [24,34]. This suggests that high-molecular-weight polysaccharide structures may be more conducive to activating immune-related signaling pathways. (Table 1).

### 2.3. Self-Assembled Structure

**Figure 1:**
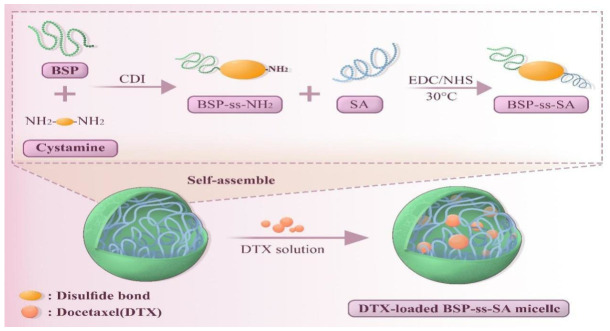
The synthesis of DTX-loaded BSP-ss-SA.

Polysaccharide bioactivity is tightly linked to molecular weight, chemical structure, and higher-order structure, with self-assembly being pivotal for constructing the latter. Regulated by intermolecular forces and environmental cues, BSP self-assembles into a characteristic triple-helix conformation that underpins its unique bioactivity and stability [9]. Congo red assays are commonly used to detect triple-helix structures but suffer from false positives/negatives, necessitating complementary characterization techniques [35,36]. Functionally, BSP’s triple-helix can further assemble into nanoscale ordered structures, showing promise in immunomodulation and anti-infection [19,37]. Its abundant hydroxyl groups confer hydrophilicity, but grafting hydrophobic moieties (e.g., stearic acid) enables self-assembly into core-shell nanomicelles in water. These micelles efficiently load water-insoluble drugs to enhance solubility and bioavailability [38]. For example, Liu et al. [39] developed a pH/redox-dual-responsive BSP-ss-SA copolymer that forms ~125 nm micelles, encapsulates docetaxel, and inhibits HepG2/4T1 proliferation via tumor microenvironment-triggered release (Figure 1). 

BSP self-assembly also drives fibrous structures, with woven fabrics, therefore, holding wound repair potential [19,37]. Despite progress in elucidating assembly mechanisms, resolving precise spatial structures remains challenging. Future research into precise techniques for correlating assembly processes with higher-order structures is critical for advancing BSP’s biomedical applications.

## 3. Chemical Modification of *Bletilla striata* Polysaccharide

Chemical modification of BSP refers to the process of modifying its molecular structure via chemical methods to optimize its physicochemical properties and biological activities. Natural BSP has limitations such as limited activity under neutral conditions and single functionality; modification aims to overcome these limitations and expand its application potential in the biomedical field.

### 3.1. Carboxymethylation Modification

**Figure 2:**
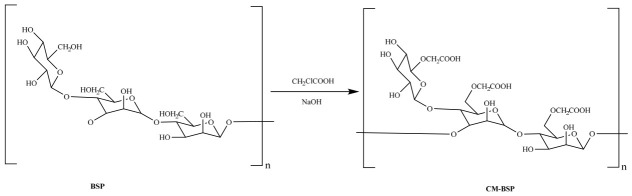
The synthetic route of CM-BSP.

Carboxymethylation is a widely used polysaccharide chemical modification technique, which introduces carboxymethyl groups at specific hydroxyl sites of polysaccharide chains to convert neutral polysaccharides into negatively charged molecules, thereby altering their physicochemical and biological properties. It is favored for its simplicity, low cost, and low toxicity [40]. Wang et al. [41] modified BSP via etherification to obtain carboxymethylated BSP (CM-BSP), with carboxymethyl substitution mainly at C-6, C-4, and C-2 (Figure 2). CM-BSP, carrying negative charges, forms water-insoluble polyelectrolyte complexes with positively charged chitosan (CS) via electrostatic interactions. The composite films from these complexes exhibit drug sustained release ability, reducing administration frequency and enhancing local drug concentration, and show great potential for developing new oral ulcer therapies due to their low biotoxicity.

Carboxymethylation primarily occurs at the C-6, C-4, and C-2 positions of BSP. In this study, when the degree of substitution (DS) of CM-BSP reached 0.8–1.2, the water solubility of CM-BSP increased by 40–60% compared to pristine BSP, and its emulsifying stability was enhanced by 25–35% under the experimental conditions [40]. This modification-induced property improvement enables CM-BSP to be applied as a high-performance stabilizer in dairy products (e.g., yogurt) to prevent phase separation during storage.

### 3.2. Graft Copolymerization

Graft copolymerization modifies polysaccharides via covalently attaching functional side chains through esterification or amidation, thereby endowing them with novel physicochemical and biological properties to expand their biomedical application scope. For instance, a study successfully synthesized a redox-sensitive stearic acid-grafted BSP copolymer (BSP-ss-SA). This copolymer self-assembled into micelles with an average diameter of (106 ± 4.36) nm; in the presence of 10 mmol/L dithiothreitol (DTT), micelle size increased significantly, confirming redox sensitivity, which is critical for tumor-targeted drug release. Evaluations of docetaxel loading and encapsulation efficiency further verified its potential as a drug delivery carrier [42].

### 3.3. Polysaccharide–Trace Element Complexes

Polysaccharide–trace element complex modification constructs synergistic complexes via coordination/covalent bonding between polysaccharide functional groups and essential trace elements. This modifies BSP’s spatial structure, enabling trace element–polysaccharide synergy, enhancing trace element bioavailability, reducing toxicity, and boosting BSP’s inherent bioactivity for expanded nutritional and disease prevention applications.

Xu et al. [43] synthesized BSP-Se, BSP-Zn, and BSP-Fe. At equal concentrations, these complexes exhibited stronger free radical scavenging (enhanced antioxidant activity) and α-glucosidase inhibition than pristine BSP, showing hypoglycemic/diabetes prevention potential. Compared to inorganic supplements, these complexes improve bioavailability, reduce toxicity, and integrate polysaccharide and trace element activities, possibly generating novel functions [44], serving as safe, efficient, multifunctional nutritional supplements. Jiang et al. [45] prepared selenylated BSP (Se-BSP). In the mouse model established in this study, at an oral administration dose of 150 mg/kg, Se-BSP with a higher molecular weight exhibited improved antioxidant activity and enhanced efficacy in alleviating liver fibrosis.

### 3.4. Cross-Linking Modification

Cross-linking modification constructs 3D networks between polysaccharide chains or with other biopolymers via covalent/non-covalent bonds, optimizing physicochemical properties and endowing bioactivities to expand biomedical applications. Yang et al. [46] cross-linked oxidized BSP and chitosan into hydrogels. As a *Lactobacillus plantarum* carrier, the hydrogel protects bacteria and exerts antibacterial activity, significantly promoting full-thickness skin wound healing by maintaining moisture, upregulating VEGF, inhibiting inflammation, accelerating collagen deposition, and reducing scarring. Zhang et al. [47] synthesized a dual-dynamic cross-linked hydrogel via one-pot reaction using BSP as the backbone, polyvinyl alcohol–borax, and tannic acid. It exhibits enhanced mechanical properties, self-healing, adhesion, antibacterial, antioxidant, and hemostatic abilities, addressing wound hemostasis and adhesion. Liu et al. [48] prepared UV cross-linked hydrogel patches from methacryloyl-BSP and gelatin. Leveraging BSP’s macrophage affinity, it regulates macrophage phenotype, inhibits NLRP3 inflammasome, and promotes diabetic wound healing via the TGF-β1/Smad2 pathway.

### 3.5. Phosphorylation Modification

Structural modification of natural polysaccharides and their complexes via chemical derivatization represents a crucial approach to regulating their biological activities, physicochemical properties, and functional characteristics. Among various modification strategies, phosphorylation modification stands out as a typical one. By introducing phosphate groups, it can alter the charge distribution, spatial conformation, and intermolecular interactions of polysaccharides, thereby significantly enhancing their bioavailability and functional diversity [49]. For instance, after phosphorylation, the scavenging efficiency of Hedysarum polybotrys polysaccharides against ABTS^+^ and DPPH· in vitro increased by 35–42% compared with the unmodified polysaccharides, showing a dose-dependent manner [50]. For BSP, phosphorylation modification may endow it with novel biological activities by regulating the flexibility and hydrophilicity of its molecular chains. Recent studies have confirmed that phosphorylated BSP exhibited a hydroxyl radical (·OH) scavenging rate of 80% at a concentration of 4 mg/mL, suggesting its potential application in the development of antioxidants and anti-inflammatory drug carriers [31]. These findings reveal that phosphorylation modification can enhance the antioxidant activity of BSP, particularly its scavenging capacity against hydroxyl radicals, which provides a theoretical basis for the structural modification and functional development of polysaccharide compounds.

### 3.6. Acetylation Modification

Acetylation of polysaccharides induces the extension of their branches, thereby exposing more hydroxyl groups, which in turn enhances water solubility and biological activity [51]. Acetylated BSP (acetylated BSP) can serve as a component for fabricating novel biomaterials. For instance, when blended with polylactic acid (PLA) to prepare composite materials, it enhances the glass transition temperature of the materials and improves their application performance [52].

### 3.7. Cholesterylation Modification

The cholesterylation of BSP refers to the introduction of cholesterol groups into BSP molecules via chemical reactions regulated by catalysts, using cholesterol succinate as the modifier. This process converts BSP from a natural water-soluble polysaccharide into an amphiphilic polymeric material with both hydrophilic backbones and hydrophobic domains, and the degree of substitution can be regulated by adjusting the dosage of cholesterol succinate. Its function is to improve the drawbacks of natural BSP, such as high water solubility, easy backbone erosion, poorly controlled release performance, and difficulty in encapsulating water-insoluble drugs, while endowing it with the self-assembly capability to expand its application as a drug carrier [53]. For instance, the conjugation of cholesterol succinate to BSP enhances its amphiphilicity. The degree of substitution of cholesterol succinate–BSP is 3.2%, and the critical micelle concentration (CMC) of the modified BSP is 0.001 mg/mL, indicating that it can self-assemble into nanoparticles in aqueous solution. Additionally, since cholesterol is an endogenous substance, the modified material maintains good biocompatibility, laying a foundation for subsequent drug-loading studies [54].

## 4. Biomedical Applications of *Bletilla striata* Polysaccharide

**Figure 3:**
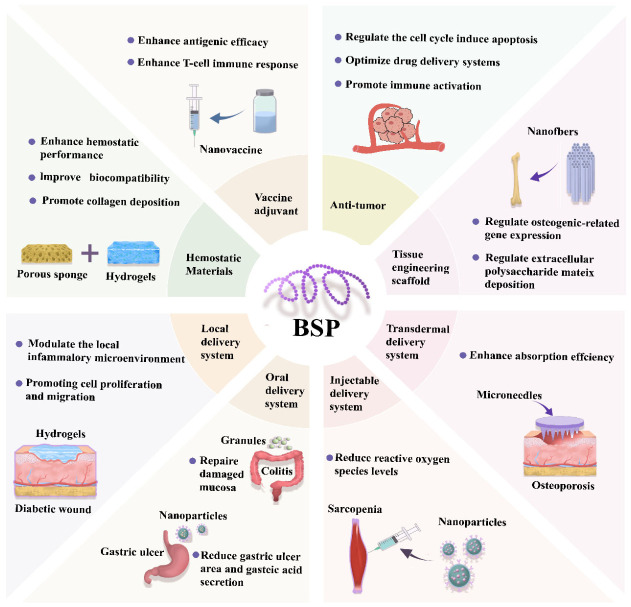
Applications of *Bletilla striata* Polysaccharide in biomedicine.

BSP has wide applications in the biomedical field. As a biomedical material, its sponges and hydrogels can stop bleeding and promote wound healing, and its scaffolds can assist tissue regeneration. In drug delivery, local, oral, injectable, and transdermal systems can improve drug efficacy and enable targeted therapy. It also possesses immunomodulatory and antitumor effects, showing multi-dimensional anticancer abilities and enhancing vaccine antigen effects, demonstrating great application potential (Figure 3).

### 4.1. Biomedical Materials

#### 4.1.1. Hemostatic Materials

Hemostatic materials prepared using BSP as the main component mostly have high-porosity porous structures, with hemostatic, anti-inflammatory, antibacterial, and high liquid-absorption properties. They enhance coagulation by shortening activated partial thromboplastin time (APTT), prothrombin time (PT), and thrombin time (TT) and increasing fibrinogen content, promoting wound healing. Common forms include sponges, hydrogels, and aerogels.

**Figure 4:**
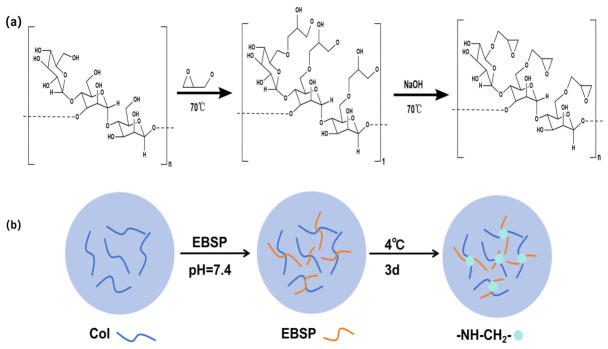
(**a**) The epoxidation of *Bletilla striata* polysaccharide. (**b**) The cross-linking reaction between epoxidized *Bletilla striata* polysaccharide and collagen.

BSP sponges (physical processing/polymer blending) form porous scaffolds that facilitate wound gas exchange and exudate removal, with structure bioactivity synergy making them ideal tissue engineering dressings [19,55,56]. Zhai et al. [57] confirmed BSP’s procoagulant potential via APTT/PT/TT shortening and fibrinogen elevation. Zhu et al. [58] developed a BSP–zeolite composite sponge that showed robust in vitro/in vivo hemostasis, mitigated zeolite-induced thermal effects, and promoted healing via BSP’s anti-inflammatory activity. Tang et al. [7] activated the primary hemostatic component of *Bletilla striata*—BSP—using epoxy groups to produce epoxidized *Bletilla striata* polysaccharide (EBSP). Subsequently, EBSP was crosslinked with collagen under alkaline conditions to fabricate a novel hemostatic material: epoxidized BSP crosslinked with a collagen hemostatic sponge (Figure 4). Compared to medical gauze, this sponge demonstrated a shorter hemostatic time (26.75 ± 2.38 s) and reduced blood loss (0.088 ± 0.051 g) in a rat liver injury hemostasis model. In a rat tail amputation hemostasis model, it also exhibited a shorter hemostatic time (47.33 ± 2.05 s) and decreased blood loss (0.330 ± 0.122 g). He et al. [59] developed a BSP-Cu-PLE sponge that enabled full-stage wound healing, with superior water absorption and antioxidant/biocompatible/biodegradable properties, and enhanced hemostasis via collagen deposition/angiogenesis.

BSP hydrogels also excel: Zhang et al. [47] prepared dual-dynamic bond cross-linked hydrogels that significantly improved biocompatibility/antibacterial/antioxidant properties for acute bleeding control; Xiang et al. [60] designed a BSP-g-PAA/PVADN hydrogel that exhibited low hemolysis and efficient coagulation in rat liver bleeding/mouse tail amputation models; Hao et al. [61] synthesized a KGM-BSP hydrogel (BDDE cross-linker) that required no modification, removed residual BDDE, preserved structure/bioactivity, and regulated inflammation/cell migration; Gao et al. [62] fabricated BSP/chitosan/β-glycerophosphate sodium hydrogels that overcame BSP dissolution-induced defects, sustained the release of SDSS, and accelerated the healing of mouse wounds.

Yan et al. [27] developed an oxidized BSP (ORBPS)/PVA aerogel that integrates antibacterial/procoagulant functions, thus enhancing healing via inflammation inhibition/angiogenesis/epithelialization. Yang et al. [63] developed a bilayer BSP-CMCS dressing (BSP-PNS@CMCS) that achieves hemostasis (inner BSP: red blood cell adhesion/coagulation shortening) and angiogenesis (outer PNS-loaded CMCS), thereby reducing rat liver bleeding.

#### 4.1.2. Tissue Engineering Scaffolds

Tissue engineering scaffolds using BSP as a key natural polymer leverage its tunable biodegradation, excellent cell affinity, and customizable mechanical properties to form 3D structures. These scaffolds support cell growth by providing a favorable microenvironment, with promising applications in bone tissue repair. For instance, in a rat alveolar bone-derived osteoblast model, BSP promotes bone regeneration and mineralization by regulating extracellular matrix deposition and osteogenesis-related gene expression [64].

### 4.2. Drug Delivery Systems

#### 4.2.1. Local Delivery Systems

Local delivery systems deliver BSP directly to the lesion via specific carriers. These systems allow drugs to act directly at the lesion, reducing systemic toxicity and achieving targeted therapy for local diseases by regulating the local microenvironment and promoting tissue repair, with the advantages of direct onset and concentrated efficacy.

Zhao et al. [65] demonstrated that BSP’s local lesion action inhibits NLRP3 inflammasome-mediated inflammation, promotes angiogenesis/tissue repair, avoids normal tissue toxicity, and accelerates diabetic wound healing. For system construction, Li et al. [66] used biocompatible Carbomer 940 and carboxymethyl chitosan (CMC) to prepare hierarchically porous BSP composite hydrogels; these hydrogels maintain wound microenvironment stability via high water retention [67,68], promote L929 fibroblast proliferation/migration, and support diabetic wound therapy.

Subsequently, Hu et al. [69] prepared B/TF hydrogels (BSP, borax, TA/Fe^3+^) that exhibit photothermal antibacterial/antioxidant activity in diabetic wounds: photothermal heating inhibits bacteria, TA/Fe^3+^ scavenges free radicals, and BSP’s anti-inflammatory properties synergize to facilitate repair. Chen et al. [67] integrated BSP into waterborne polyurethane to prepare low-cost, easily fabricated wound hydrogels; within these hydrogels, BSP regulates inflammation/promotes cell activity, while polyurethane enables a sustained release, aiding clinical translation.

**Figure 5:**
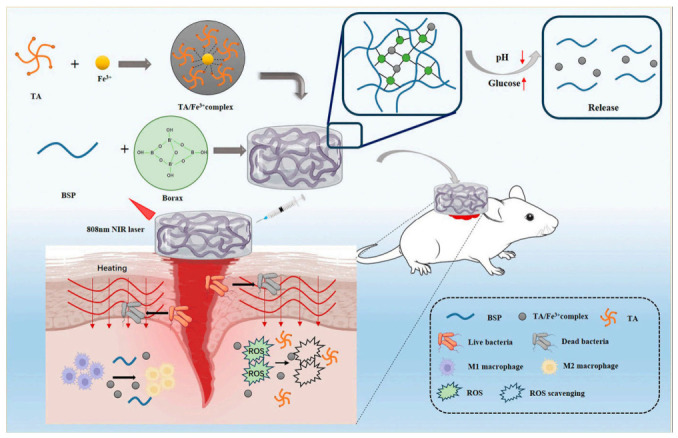
The application mechanism of the intelligent hydrogel system based on BSP and tannic acid (TA)/Fe^3+^ complex in wound therapy [69].

Hu et al. [70] prepared BSP/berberine (BER)/Carbomer 940 hydrogels that show excellent biocompatibility, antibacterial/anti-inflammatory/ antioxidant properties, with 2% BSP-BER (at a 1:40 mass ratio) most effectively accelerating diabetic wound healing (Figure 5). Zhao et al. [71] constructed a CuO@BER/BH hydrogel (oxidized BSP-HACC Schiff base matrix, BER-loaded nanoparticles) that protects wounds via physical barriers, NIR-promoted BER release, and multi-effects (antibacterial/anti-inflammatory/antioxidant) for targeted, low-systemic toxicity diabetic infected wound healing.

#### 4.2.2. Oral Delivery Systems

Oral delivery systems transport BSP into the body via oral administration using suitable carriers. These systems improve drug stability and absorption efficiency in the gastrointestinal tract, achieve targeted therapy for gastrointestinal diseases via mechanisms such as delayed gastric emptying, mucosal adhesion, or targeted release, and combine convenience with targeting.

Currently, diverse BSP-based delivery systems have shown potential for oral applications. Oligomeric proanthocyanidin/BSP/chitosan composite microspheres function as oral sustained release carriers, enhancing drug absorption via delayed gastric emptying and intestinal mucosal adhesion [72]. For gastric ulcer treatment, Li et al. [73] investigated BSP–omeprazole (OME) nanoparticles (OME-BSP NPs); these not only reduce ulcer area, inhibit gastric acid secretion, and reverse gastric tissue damage/apoptosis but also improve OME stability and absorption in rat stomachs, enabling targeted ulcer therapy.

**Figure 6:**
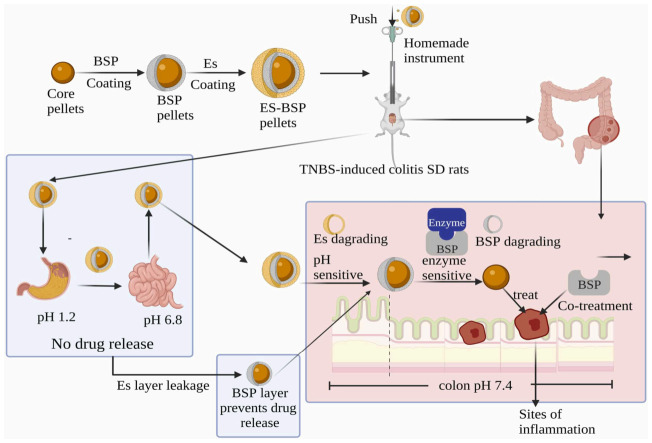
Colon-targeted drug delivery system of *Bletilla striata* polysaccharide-based double-layer coated pellets for the treatment of colitis [74].

Notably, Sun et al. [74] innovatively employed BSP as a film-coating material to develop dual pH/enzyme-sensitive Gegenqinlian pellets for ulcerative colitis. BSP repairs damaged colonic mucosa, exerts synergistic therapeutic effects, and prevents adverse reactions from premature release of single pH-sensitive pellets in the small intestine, confirming BSP’s key role in boosting drug efficacy and its promise as a novel adjuvant for ulcerative colitis treatment (Figure 6).

#### 4.2.3. Injectable Delivery Systems

Injectable delivery systems transport BSP into the body via intravenous, intramuscular, or other injection routes using chemically modified or composite carriers. These systems improve drug bioavailability and reduce toxic side effects by optimizing drug-loading performance, enhancing targeting, or achieving controlled release, and are suitable for the treatment of liver targeting, tumor chemotherapy, sarcopenia, and other diseases.

For example, Zhang et al. [75] fabricated stearic acid-modified BSP nanomicelles loaded with docetaxel: In this reported system, the stearic acid-modified BSP nanomicelles showed a substitution degree of 12.94%, a critical aggregation concentration of 3.09 μg/mL, encapsulation efficiency of 81.11%, and drug loading capacity of 9.13%. Intravenous injection promoted liver, spleen, and tumor accumulation (hydrophobic segment/EPR effect), avoiding hepatic first-pass metabolism. Ma et al. [76] similarly encapsulated silymarin in stearic acid-modified BSP nanoparticles; intravenous delivery achieved a liver-specific accumulation via the reticuloendothelial system (RES) uptake, avoiding gastrointestinal hydrolysis. Both studies confirm that the substitution degree regulates drug-loading for liver/tumor-targeted intravenous delivery.

Conversely, Liang et al. [77] prepared intramuscular BSP–hydroxyapatite (HAP) formulations (13.6% loading, HAP BET = 60.76 m^2^/g). Macrophage endocytosis triggers the BSP release in acidic lysosomes (pH 3) for controlled release, reducing LPS-induced ROS by 19% and downregulating inflammation to treat sarcopenia.

#### 4.2.4. Transdermal Delivery Systems

Transdermal delivery systems use BSP as an active ingredient or carrier to enable BSP to cross the skin barrier and be released in a targeted manner in local skin or subcutaneous tissue via specific formulation design and technical means, achieving biological effects such as wound healing promotion, anti-inflammatory, and antibacterial effects. These systems need to balance the retention of BSP’s biological activity and skin penetration efficiency.

**Figure 7:**
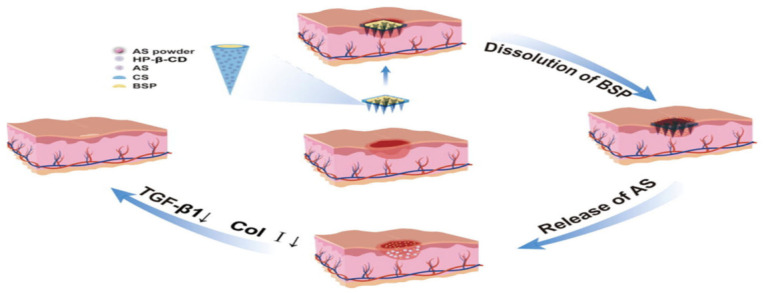
The mechanism of wound repair and anti-scarring by *Bletilla striata* polysaccharide-based nanoparticles in combination with asiaticoside [81].

Hu et al. [78] developed soluble BSP microneedles with excellent moldability and demoldability; these penetrate the stratum corneum to form drug channels, enabling a sustained release via dissolution in skin interstitial fluid, with promising transdermal application potential. Building on this, Wang et al. [79] designed risedronate sodium (RIS)-loaded BSP microneedles (RIS-BMN) to address oral RIS limitations in postmenopausal osteoporosis treatment. In the in vitro tests of this study, RIS-BMN showed 3-fold and 2.6-fold higher in vitro absorption than the RIS solution and commercial patches, as well as minimal skin irritation (resolving within 12 h), and anti-osteoporotic effects comparable to oral RIS, improving treatment compliance. Yang et al. [80] prepared chitosan/BSP composite microneedles (with in situ-generated silver nanoparticles in the pores). In the antibacterial assays of this work, this formulation boosted antibacterial efficacy by 75% compared with microneedles without silver nanoparticles and penetrated the skin barrier to disrupt biofilms for infected wound treatment. Lv et al. [81] developed bilayer CS-AS-BSP MNs (BSP/chitosan asiaticoside [AS]-containing) that use upper-layer BSP (hemostasis/anti-inflammation and collagen promotion) and sublayer AS (inhibits excessive collagen/TGF-β1 and reduces fibroblasts/type I collagen [Col I]) for scar-free wound healing (Figure 7).

### 4.3. Immunomodulation and Antitumor Effects

#### 4.3.1. Multi-Dimensional Antitumor Mechanisms and Applications

BSP and its derivatives exert antitumor effects via multi-target synergy, including cell cycle regulation, apoptosis induction, immune activation, and drug delivery optimization. At the cellular level, BSP disrupts tumor cell behavior; animal studies confirm its activity; BSP suppresses HepG2 xenograft growth and reshapes the CT26/H22 dual-tumor microenvironment to enhance antitumor immunity via TLR4/NF-κB activation [82,83]. BSP-based nanocarriers excel in drug delivery: Li et al. [84] constructed BSP–paclitaxel nanoparticles that boost MKN45 gastric cancer cell antiproliferation via enhanced tumor drug accumulation and synergize with 5-fluorouracil (5-FU) for chemosensitization. In the H22 liver cancer mice, BSP nanocarriers reduce tumor size and weight, increase inhibition rates, and improve liver, spleen, and thymus immune function [85]. Structural modification enhances targeting: Wang et al. [86] prepared folate-modified BSP–stearic acid (FA-BSP-SA) micelles loaded with doxorubicin (Dox@FA-BSP-SA); BSA binding to the micelles reduces antitumor efficacy, while unbound micelles effectively inhibit 4T1 cell proliferation (** *p* < 0.01 and *** *p* < 0.001). Liu et al. [87] developed a pH-redox dual-responsive BSP copolymer for tumor microenvironment-specific drug release. Low-molecular-weight BSP (low-temperature extraction) shows stronger antitumor potential: Liu et al. [5] found it exhibits higher anticancer activity in H22 liver cancer-bearing mice, likely due to improved bioavailability. Additionally, phenanthrene compounds from *Bletilla striata* exert selective cytotoxicity against HL-60 leukemia and A549 lung cancer cells, expanding BSP derivative research [88].

#### 4.3.2. Enhancing Antigen Efficacy as a Vaccine Adjuvant

Since the 1930s discovery of herbal polysaccharides’ immunomodulatory, antiviral, and antitumor activities, they have become key natural immunomodulators due to low toxicity, minimal side effects, definite efficacy, and abundant resources [24,30], regulating humoral/cellular immunity via enhanced cytokine expression, CD4+/CD8+ T cell activation, and NK cell activity [89]. As a pharmaceutical excipient, BSP has bioadhesiveness and biodegradability, showing potential in vaccine delivery (e.g., microneedle vaccines) for transdermal model protein antigen delivery, avoiding injection discomfort/risk and improving compliance [90]. SP also enables self-adjuvant nanovaccines (integrating adjuvant and antigen), leveraging its immunomodulatory properties and nanocarriers’ advantages to enhance antigen delivery to APCs and immune responses [91]. In addition, BSP has applications in vaccine carriers. Zhou et al. [90] successfully prepared BSP microneedles (BMN) that outperform hyaluronic acid/polyvinyl alcohol counterparts in mechanical properties and stability, with good biocompatibility, low skin bacterial permeability, and minimal irritation, showing promise as efficient vaccine delivery vehicles.

## 5. Application of *Bletilla striata* Polysaccharide in the Food Industry

**Figure 8:**
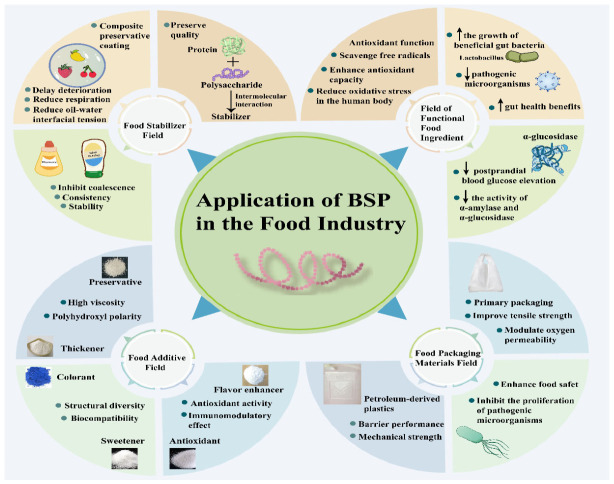
*Bletilla striata* Polysaccharide: applications in the food industry.

BSP acts as a natural stabilizer and thickener for processed foods, a composite coating for fresh produce, a functional food additive, and a bio-based packaging component (Figure 8). Its unique physicochemical properties and biological activities make it a versatile candidate for food industrial innovation, aligning with the trend of “natural, functional, and sustainable” food development.

### 5.1. Field of Food Stabilizer

In food formulations, the stabilization performance of BSP is intrinsically linked to its glucomannan backbone structure, which consists of β-(1→4)-linked D-Man*p* and D-Glc*p* residues with a molar ratio of 1:1 to 4:1, a molecular weight range of 10^4^–10^5^ Da, and a low degree of branching at the O-6 position of glucose residues, all of which dictate its intermolecular interactions, rheological behavior, and interfacial activity in complex food matrices [3,9]. Unlike generic polysaccharide stabilizers such as xanthan gum, guar gum, and carboxymethyl cellulose, BSP exhibits unique shear-thinning behavior, high water-holding capacity, and excellent compatibility with milk proteins, starch, and other common food hydrocolloids, making it a versatile natural stabilizer, thickener, and emulsifier that aligns with the global clean-label food development trend [30,92].

The stabilization mechanism of BSP in food systems is driven by three synergistic molecular effects that are fully characterized in BSP-specific studies, rather than generic polysaccharide theories. First, the abundant hydroxyl groups along the BSP backbone form extensive intra- and intermolecular hydrogen bonds with water molecules, food proteins, and other hydrocolloids, constructing a continuous viscous network in the aqueous phase that increases apparent viscosity, restricts molecular movement, and inhibits particle sedimentation, oil droplet creaming, and phase separation during storage [32,93]. Second, the flexible linear chains of BSP can rapidly adsorb and anchor at the oil–water interface, reducing the interfacial tension of oil-in-water emulsions from 18.6 mN/m to 9.2 mN/m within 30 min, and forming a dense, viscoelastic interfacial film that prevents droplet coalescence and flocculation via steric hindrance [31]. Third, chemically modified BSP with negatively charged groups (such as carboxymethyl and phosphate groups) further enhances the stability of dispersed systems through the combined effect of electrostatic repulsion and steric hindrance, addressing the limitation of native BSP’s weak stabilization performance in high-salt and low-pH food systems [40,41].

Native BSP has been validated to exhibit excellent stabilization and thickening effects in multiple commercial food categories, with clear quantitative performance data from application tests. In fermented dairy products such as yogurt, the addition of 0.3–0.5% (*w*/*w*) native BSP increases the apparent viscosity of the product by 45–62%, reduces whey separation rate by 78% during 21 days of cold storage at 4 °C, and maintains a uniform, smooth texture without significant changes in sensory acceptability, outperforming gelatin at the same addition level [93]. In oil-in-water emulsion systems, including salad dressings and creamers, native BSP at 0.4% addition reduces the average droplet size of the emulsion from 2.8 μm to 1.1 μm, and increases the emulsifying stability index (ESI) by 28% after 30 days of storage at room temperature [31]. When compounded with xanthan gum at a mass ratio of 1:2, BSP forms a thermoreversible gel network that effectively inhibits syneresis in low-sugar jams and fruit fillings, reduces water loss by 52% after three freeze–thaw cycles, and optimizes the spreadability and texture of the products [93]. In frozen dough and frozen dessert systems, BSP at 0.2% addition reduces ice crystal growth by 63% during frozen storage, lowers thawing loss by 41%, and maintains the soft texture of the dough after baking, addressing the quality deterioration of frozen foods caused by ice crystal recrystallization [3]. In plant-based beverage systems, BSP effectively prevents the sedimentation of protein and dietary fiber particles, extends the physical shelf life of the product by more than 60 days at room temperature, and has no adverse effect on the flavor and color of the beverage [30].

Chemical modification is a critical strategy to further enhance the stabilization performance of BSP and expand its application scope in harsh food processing conditions, with multiple BSP-specific modification methods validated for industrial feasibility. Carboxymethylation is the most widely studied modification for BSP stabilization optimization: when the degree of substitution (DS) of carboxymethylated BSP (CM-BSP) reaches 0.8–1.2, its water solubility increases by 50–60% compared with native BSP, emulsifying activity increases by 32%, and it maintains stable structural and functional properties after high-temperature sterilization at 121 °C for 30 min, overcoming the key limitations of native BSP including poor heat resistance and low solubility in neutral and acidic aqueous systems [41,42]. Acetylated BSP also shows improved emulsifying performance, with a 25% higher ESI than native BSP in acidic beverage emulsions, making it suitable for acidic fruit juice and sports drink systems [51]. Phosphorylated BSP exhibits an enhanced electrostatic stabilization effect in high-salt food systems, maintaining 85% of its emulsifying stability in the presence of 200 mmol/L sodium chloride, while native BSP loses more than 60% of its stabilization performance under the same conditions [31,49].

Compared with commercially available natural food stabilizers, BSP has unique advantages, including excellent biocompatibility, pH tolerance, and synergistic effects with other hydrocolloids, while its current industrial application is limited by the lack of large-scale extraction and purification processes and insufficient long-term safety data in complex food matrices [3,23]. Future research on BSP as a food stabilizer should focus on three core directions: the establishment of low-cost, green extraction and modification processes for industrial-scale production, the systematic evaluation of its safety and stability in different food processing and storage conditions, and the development of compound stabilizer formulations based on BSP to meet the diversified needs of the food industry.

### 5.2. Field of Food Additives

BSP, a class of natural biomacromolecules, exhibits versatile functionality as a food additive, attributed to its inherent high viscosity, polyhydroxyl polar structure, structural diversity, and excellent biocompatibility. Beyond its physicochemical advantages, BSP also possesses prominent biological activities, most notably antioxidant and immunomodulatory effects [3,94]. These combined properties make BSP a promising candidate for formulating functional foods, as it can impart health-promoting attributes to end products while fulfilling the technical roles of a food additive [30,95].

In practical applications, BSP can be used as a natural antioxidant additive in oil-rich foods (e.g., edible oils and fried snacks) to inhibit lipid oxidation. Research has shown that BSP extracted via deep eutectic solvents (DESs) exhibits significant scavenging activity against DPPH· and ABTS+ radicals, with a ferric-reducing antioxidant power comparable to synthetic antioxidants like butylated hydroxyanisole (BHA) at appropriate concentrations [96,97]. Additionally, BSP’s emulsifying property enables it to disperse uniformly in food matrices, ensuring consistent antioxidant efficacy throughout the product’s shelf life.

### 5.3. Field of Functional Food Ingredients

As a typical “medicine–food homology” component, BSP has significant potential as a functional food ingredient, with biological activities closely related to food nutrition and human health. Its application in functional foods is supported by rigorous in vitro and in vivo studies, adhering to the requirement of “food relevance” in food chemistry.

#### 5.3.1. Antioxidant Function

BSP’s antioxidant activity is derived from its structural characteristics—such as branched chains exposing more hydroxyl groups and α-glucopyranose conformation—which enable it to scavenge free radicals [9,29]. In functional beverage development, adding BSP can enhance the product’s antioxidant capacity, helping to reduce oxidative stress in the human body [92].

#### 5.3.2. Gut Health Regulation

BSP exhibits prebiotic potential by promoting the growth of beneficial gut bacteria (e.g., Bifidobacterium and Lactobacillus) and inhibiting pathogenic microorganisms. In vitro anaerobic fermentation experiments have shown that BSP can be degraded by gut microbiota to produce short-chain fatty acids (SCFAs), which regulate intestinal pH and improve intestinal barrier function [98]. This supports its application in functional dairy products (e.g., probiotic yogurt) to synergistically enhance gut health benefits.

#### 5.3.3. Blood Glucose Management

BSP has been shown to inhibit the activity of α-amylase and α-glucosidase, key enzymes involved in carbohydrate digestion, thereby slowing postprandial blood glucose elevation [95]. In functional cereal products (e.g., whole-grain biscuits), BSP can be added as a functional ingredient to assist in blood glucose control, with relevant in vivo studies confirming its safety and efficacy at food-grade dosages [43].

### 5.4. Field of Food Packaging Materials

As a sustainable alternative to petroleum-derived food packaging plastics, BSP exhibits outstanding application potential in bio-based packaging and edible coating systems, relying on its inherent film-forming capacity, biocompatibility, biodegradability, and intrinsic antioxidant/antibacterial activities [3,16]. With a linear glucomannan backbone of β-(1→4)-linked D-Man*p* and D-Glc*p* residues and abundant reactive hydroxyl groups, BSP can form transparent, flexible films via scalable solution casting, and can be compounded with chitosan, starch, or natural active agents to tune its functional properties for food packaging applications [15,31].

Food-grade BSP-based packaging materials are mainly fabricated via three industrial compatible systems: BSP–chitosan cross-linked blends, BSP–starch cost-effective composites, and BSP–active agent (essential oils, plant extracts) functional systems, with electrospun BSP nanofiber membranes also developed for high-breathability fresh food packaging [3,13]. These BSP-based materials meet industrial packaging requirements with clear BSP-specific quantitative performance: a tensile strength of 18.6–27.3 MPa (comparable to commercial LDPE), a water vapor transmission rate of 2.1 × 10^−10^–3.8 × 10^−10^ g·mm/(cm^2^·s·Pa), an oxygen transmission rate of 0.8–1.5 cm^3^/(m^2^·d·atm), 92% biodegradation in soil within 28 days, and a DPPH radical scavenging rate of 68–83% with a 72–89% inhibition rate against *E. coli* and *S. aureus* [9,19,31].

BSP-based packaging and coatings show significant preservation efficacy with validated shelf-life extension data. For fresh fruits and vegetables, BSP–essential oil composite coatings reduce the respiration rate by 47% and delay decay by 12–15 days for mangoes and strawberries [99]. For chilled pork, BSP–chitosan films reduce total volatile basic nitrogen content by 58% and extend shelf life from 3 days to 9 days at 4 °C [31]. For oil-rich foods, BSP films inhibit peroxide value rise by 65%, extending edible oil shelf life by 25 days at room temperature [3].

Compared with common chitosan/starch-based packaging, BSP-based materials have unique advantages of better moisture retention, inherent bioactivity, and no adverse effect on food sensory properties, while their industrial application is limited by high extraction cost and poor water resistance in high-humidity environments. Future research should focus on green low-cost extraction processes, water resistance optimization via food-grade cross-linking, and systematic food safety assessment to promote its industrial translation [3,20].

## 6. Conclusions and Prospects

### 6.1. Core Conclusions

This review systematically collates the latest research progress of *Bletilla striata* polysaccharide (BSP) from 2016 to 2025, establishing a complete logical framework from its glucomannan-based structural characteristics, full-spectrum chemical modification strategies, to expanding applications in both biomedicine and the food industry. Compared with previously published reviews, the core contributions of this work lie in the systematic analysis of BSP’s multi-dimensional structure–activity relationship covering primary structure to higher-order self-assembled triple-helix conformation, the full-spectrum classification and mechanism interpretation of seven major chemical modification strategies for BSP, and the first comprehensive and parallel integration of BSP’s research advances in the dual core fields of biomedicine and the food industry, filling the gaps of single-field focus and fragmented narrative in existing reviews. As a natural glucomannan from a medicine–food homology herb, BSP exhibits unique and irreplaceable advantages over other widely used natural polysaccharides, including chitosan, hyaluronic acid, sodium alginate, and konjac glucomannan. It has excellent water solubility and neutral pH compatibility with extremely low cytotoxicity, overcoming the dissolution limitation of chitosan under neutral conditions, while its flexible linear backbone and abundant modifiable hydroxyl sites endow it with stronger film-forming ability, tissue adhesiveness, and chemical designability than konjac glucomannan and sodium alginate. Most notably, BSP has an integrated dual role of bioactive functional component and structural material, inherently possessing hemostatic, anti-inflammatory, antioxidant, and pro-regeneration pharmacological activities that inert polysaccharide excipients such as hyaluronic acid do not have, and its official medicine–food homology attribute also gives it inherent regulatory compliance advantages in food applications that non-edible polysaccharide materials cannot match.

### 6.2. Critical Personal Remarks

Through in-depth collation and analysis of nearly 10 years of BSP research, we put forward the following critical academic judgments on the current development status of this field. At present, BSP-related research is facing several core bottlenecks that restrict its high-quality development and practical translation. First, the research on the structure–activity relationship of BSP is severely fragmented and methodologically inconsistent; the lack of unified standard protocols for extraction, purification, and structural characterization leads to significant heterogeneity of the BSP raw materials used in different studies, making it difficult to cross-verify results between research teams, and most reported structure–activity correlations are only phenomenological descriptions rather than molecular-level causal verification. Second, the existing chemical modification research has obvious blindness; most studies only focus on the improvement of a single in vitro performance without targeted molecular design for specific application scenarios, and most modified derivatives lack systematic in vivo efficacy and biosafety evaluation, resulting in extremely low translational value. Third, there is a serious disconnection between application research and industrial/clinical translation; most BSP-based biomaterials have only been verified in small animal models without large animal experiments and long-term in vivo safety data, and the lack of unified industrial quality control standards and low-cost, scalable extraction processes also severely restrict its large-scale application in the food industry. Fourth, the research on the molecular mechanism of BSP’s biological activity is superficial and highly homogeneous; most studies only repeat the verification of known activities, while the research on precise molecular targets, specific receptors, and downstream signaling pathways of BSP is extremely insufficient, which makes it impossible to realize the rational targeted design of BSP.

### 6.3. Future Perspectives

To address the above core bottlenecks, future research on BSP should focus on the following key directions. First, establish unified standard protocols for BSP extraction, purification, and structural characterization, and use advanced characterization techniques to carry out systematic research on multi-dimensional structure–activity relationships and establish quantitative models to realize the rational design of BSP with targeted functions. Second, develop green, efficient, and food/medical-grade chemical modification strategies closely combined with practical application scenarios and carry out complete in vivo efficacy, long-term biosafety, and pharmacokinetic evaluation for modified derivatives to lay a solid foundation for translational application. Third, accelerate the formulation of a unified quality control standard system for BSP, break through the key technologies of industrial-scale green extraction and purification to reduce production costs, and support its large-scale industrial application. Fourth, use advanced omics and molecular biology techniques to identify the specific molecular targets and action mechanisms of BSP, and expand its cross-field application potential between biomedicine and the food industry, to fully exploit the application value of this versatile natural polysaccharide.

## Figures and Tables

**Table 1:** A summary of extraction methods and structural characteristics of various *Bletilla striata* Polysaccharides.

Polysaccharide Name	Extraction Method	Monosaccharide Composition	Molecular Weight Determination Method	Molecular Weight (KDa)	Structural Characteristics	Reference
Water-soluble BSP-1	Ethanol pretreatment, hot water extraction (90 °C, 9 h), ethanol precipitation (75% ethanol, 48 h)	Man:Glc = 4.0:1.0	HPGPC-MALLS-RI	83.54	Backbone: β-(1→4)-linked D-Man*p* and D-Glc*p*; branching: none; conformation: linear	[24]
Water-soluble BSP-2	Ethanol pretreatment, hot water extraction (90 °C, 9 h), ethanol precipitation (75% ethanol, 48 h)	Man:Glc = 3.0:1.0	HPGPC-MALLS-RI	12.6	Backbone: β-(1→4)-linked D -Man*p* and D-Glc*p*; branching: none; conformation: linear	[24]
BSPF2	Acetone extraction, hot water extraction	Man:Glc:Gal = 9.4:2.6:1.0	HPGPC	235	Backbone: (1→4)-linked Man*p* and Glc*p*; branching: ~60% Glc*p* residues branched at O-6; terminal sugar: Man*p*	[25]
BSPb	Hot water extraction	Man:Glc = 1:3	HPGPC	260	Backbone: α-(1→4)-linked D-Man*p* and β-(1→4)-linked D-Glc*p*; Branching: Not reported; Conformation: Linear	[26]
RBP	Hot water extraction (100 °C, 1 h), ethanol precipitation (anhydrous ethanol, 12 h), lyophilization	Man:Glc = 2:1	GPC	820	Backbone: β-(1→4)-linked Man*p* and Glc*p*; Branching: Low degree; Conformation: Linear	[27]
pBSP	Hot water extraction (80 °C, 2 h), ethanol precipitation (95% ethanol, 12 h)	Man:Glc = 1.34:1.00	HPGPC	327.6	Backbone: β-(1→4)-linked D-Man*p*, α-(1→4)-linked D-Glc*p*, β-(1→3)-linked D-Man*p*; branching: none	[28]
pFSP	Hot water extraction (80 °C, 2 h), ethanol precipitation (95% ethanol, 12 h)	Man:Glc:Gal = 3.45:1.00:2.03	HPGPC	91	Backbone: α-(1→4)-linked D-Glc*p*, β-(1→4)-linked D-Man*p*, β-(1→3,6)-linked D-Man*p*; branching: β-(1→6)-linked D-Gal*p*; terminal sugar: β-(1→)-linked D-Man*p*	[28]
Neutral BSP-1	Alkali-assisted extraction, ethanol precipitation	Man:Glc = 3:1	GPC	269.121	Backbone: β-(1→4)-linked Man*p* and Glc*p*; branching: not reported; conformation: linear	[31]
Neutral BSP-2	Alkali-assisted extraction	Man:Glc = 3:1	GPC	57.389	Backbone: β-(1→4)-linked Man*p* and Glc*p*; branching: not reported; conformation: linear	[31]
Neutral BSP-3	Alkali-assisted extraction	Man:Glc:Gal = 6:3:1	GPC	28.153	Backbone: β-(1→4)-linked Man*p* and Glc*p*; branching: trace; conformation: linear	[31]
BSPs-A	Alkali-assisted extraction, ethanol precipitation	Man:Glc:Gal = 55.91:43.07:1.02	HPGPC	230.63	Backbone: β-(1→4)-linked Man*p* and Glc*p*; branching: low; conformation: rough lamellar	[9]
BSPs-B	Boiling water extraction, ethanol precipitation	Man:Glc:Gal = 51.30:47.55:1.16	HPGPC	402.17	Backbone: β-(1→4)-linked Man*p* and Glc*p*; branching: moderate; conformation: amorphous	[9]
BSPs-H	Hot water extraction, ethanol precipitation	Man:Glc:Gal = 60.78:37.89:1.32	HPGPC	282.91	Backbone: β-(1→4)-linked Man*p* and Glc*p*; branching: moderate; conformation: α-glucopyranose	[9]
BSPs-U	Ultrasound-assisted extraction, ethanol precipitation	Man:Glc:Gal = 61.04:38.05:0.92	HPGPC	195.83	Backbone: β-(1→4)-linked Man*p* and Glc*p*; branching: low; conformation: fragmented linear	[9]

Note: HPGPC-MALLS-RI = high-performance gel permeation chromatography–multi-angle laser light scattering–refractive index detection and GPC = gel permeation chromatography. High-performance gel permeation chromatography (HPGPC) is the synonymous term of high-performance size exclusion chromatography (HPSEC), and the two terms refer to the same chromatographic technique for polysaccharide molecular weight determination.

## Data Availability

All data used in this review are derived from publicly accessible published literature. The original data corresponding to the research results cited can be obtained through the Digital Object Identifiers (DOIs) provided in the reference list. No new experimental data were generated in this review, so no additional datasets were deposited in public repositories.

## Abbreviations

5-FU: 5-Fluorouracil; APC: Antigen-Presenting Cell; APTT: Activated Partial Thromboplastin Time; AS: Asiaticoside; BDDE: 1,4-Butanediol Diglycidyl Ether; BER: Berberine; BET: Brunauer–Emmett–Teller (Specific Surface Area); BSA: Bovine Serum Albumin; BSP: *Bletilla striata* Polysaccharide; BSPF2: Polysaccharide Fraction 2 from *Bletilla striata*; BSP-ss-SA: Redox-sensitive Stearic Acid-grafted *Bletilla striata* Polysaccharide Copolymer; BSP-Se/Zn/Fe: *Bletilla striata* Polysaccharide Selenium/Zinc/Iron Complex; BSPs-H/A/B/U: *Bletilla striata* Polysaccharides Extracted by Hot Water/Alkali/Boiling Water/Ultrasound; CM-BSP: Carboxymethylated *Bletilla striata* Polysaccharide; CMCS: Carboxymethyl Chitosan; CMC (Carboxymethyl Chitosan): Carboxymethyl Chitosan; CMC (Critical Micelle Concentration): Critical Micelle Concentration; Col I: Type I Collagen; Dox: Doxorubicin; DTT: Dithiothreitol; FA-BSP-SA: Folate-modified *Bletilla striata* Polysaccharide-Stearic Acid Copolymer; FT-IR: Fourier Transform Infrared Spectroscopy; Gal: Galactose; Glc: Glucose; GMP: Good Manufacturing Practice; GPC: Gel Permeation Chromatography; HAP: Hydroxyapatite; HPLC: High-Performance Liquid Chromatography; HPGPC: High-Performance Gel Permeation Chromatography; HPSEC: High-Performance Size Exclusion Chromatography; HWE: Hot Water Extraction; KGM: Konjac Glucomannan; LPS: Lipopolysaccharide; MALLS-RID: Multi-Angle Laser Light Scattering and Refractive Index Detection; Man: Mannose; MW: Molecular Weight; NLRP3: NOD-like Receptor Pyrin Domain-Containing Protein 3; NMR: Nuclear Magnetic Resonance; OME: Omeprazole; OME-BSP NPs: Omeprazole–*Bletilla striata* Polysaccharide Nanoparticles; ORBPS: Oxidized *Bletilla* Rhizome Polysaccharide; pBSP: Water-soluble Polysaccharide from *Bletilla striata*; pFSP: Novel Polysaccharide from *Bletilla striata* Fibrous Roots; PLA: Polylactic Acid; PNS: Panax Notoginseng Saponins; PT: Prothrombin Time; PVA: Polyvinyl Alcohol; RBP: *Bletilla striata* Polysaccharide from a Specific Source; RES: Reticuloendothelial System; RIS: Risedronate Sodium; RIS-BMN: Risedronate Sodium-Loaded *Bletilla striata* Polysaccharide Microneedles; ROS: Reactive Oxygen Species; SAR: Structure–Activity Relationship; SDSS: Sodium Danshensu; SEM: Scanning Electron Microscopy; Se-BSP: Selenylated *Bletilla striata* Polysaccharide; TEM: Transmission Electron Microscopy; TGF-β1/Smad2: Transforming Growth Factor-β1/Smad Family Member 2; TT: Thrombin Time; UAE: Ultrasound-Assisted Extraction; VEGF: Vascular Endothelial Growth Factor; Xyl: Xylose.
